# Insights into the genetic bases of differential organ size in cactus flowers: a case study in two contrasting species

**DOI:** 10.1093/aob/mcag046

**Published:** 2026-03-05

**Authors:** Isaura Rosas-Reinhold, Cristian Genaro Ramírez-Castro, Cristian R Cervantes, Gustavo Rodríguez-Alonso, Alma Piñeyro-Nelson, Ulises Rosas, Salvador Arias

**Affiliations:** Center for Genomics and Systems Biology, New York University, New York, NY 10003, USA; Jardín Botánico, Instituto de Biología, Universidad Nacional Autónoma de México, 04510 Ciudad de México, México; Posgrado en Ciencias Biológicas, Unidad de Posgrado, Ciudad Universitaria, 04510 Ciudad de México, México; Jardín Botánico, Instituto de Biología, Universidad Nacional Autónoma de México, 04510 Ciudad de México, México; Posgrado en Ciencias Biológicas, Unidad de Posgrado, Ciudad Universitaria, 04510 Ciudad de México, México; Unidad de Síntesis en Sistemática y Evolución, Instituto de Biología, Circuito Exterior s.n., Ciudad Universitaria, 04510 Ciudad de México, México; Centro de Investigación en Dinámica Celular, Instituto de Investigación en Ciencias Básicas y Aplicadas, Universidad Autónoma del Estado de Morelos, 62210 Cuernavaca, Morelos, México; Departamento de Producción Agrícola y Animal, Universidad Autónoma Metropolitana-Xochimilco, 04960 Ciudad de México, México; Jardín Botánico, Instituto de Biología, Universidad Nacional Autónoma de México, 04510 Ciudad de México, México; Jardín Botánico, Instituto de Biología, Universidad Nacional Autónoma de México, 04510 Ciudad de México, México

**Keywords:** Hylocereeae, *Disocactus*, *Disocactus eichlamii*, *Disocactus speciosus*, perianth growth, final organ size, growth evolution, floral bud transcriptomes, flower transcriptome

## Abstract

**Background and Aims:**

Developmental genetic studies in model species have unveiled genes that modulate organ size. The roles of homologues of these genes in angiosperms with complex ontogenies such as the ‘flower shoots’ of Cactaceae have not yet been analysed. Here we present histological, cellular and transcriptome analysis from flower buds in two developmental stages for two cactus species with contrasting flowers: *Disocactus speciosus* and *D. eichlamii.*

**Methods:**

Analyses of cell area, cell number and overall tepal size from floral buds were performed to determine if differential cell proliferation and expansion occurred. We also performed transcriptome analyses derived from size 1 and 2 floral buds and differential gene expression analysis, followed by KEGG enrichment.

**Key Results:**

Comparative histological and cellular analyses in *Disocactus* tepals indicate that while size 1 cells are similar in size in both species, a prolonged expansion phase takes place in *D. speciosus*. Transcriptome analyses suggest similar overall expression patterns per size, but differential expression of genes related to tepal growth were documented, like *BIG PETALp* (*BPp*), downregulated in size 1 vs size 2 in *D. eichlamii*. In this species we found *BIG BROTHER* (*BB*) was upregulated in size 1 vs size 2. In contrast, in *D. speciosus* we found two copies of *BB*, one copy upregulated and the other downregulated, as well as *NAC100*, which could be related to the final size of the flower in this species.

**Conclusions:**

This study integrates cellular and molecular data related to the development of differential organ size in Cactaceae. We found that a prolonged period of cell proliferation and expansion in *D. speciosus* tepals in contrast with *D. eichlamii* tepals seems to correlate with the final size of the flower, while transcriptome analyses point to several differentially expressed genes that should be further investigated through gene expression analyses as well as other reverse genetics approaches.

## INTRODUCTION

A central conundrum in plant evolutionary developmental biology is how organ size is controlled and whether the genetic mechanisms underlying organ growth are conserved across Embryophytes (Hong and Roeder, 2017). Organ size in plants is influenced by external environmental signals including light, nutrients and especially plant hormones (Hu *et al.*, 2003); however, cell sizes are predictable for a given species and tissue, implying that the programme controlling the growth of organs to their final form, as well as the balance between cell growth and cell division, is genetically controlled (Sablowski and Carnier Dornelas, 2014).

In angiosperms, flower size results from the balance between pollination efficiency and the energetic cost of building floral structures (Davis *et al.*, 2008). Size, together with other features like colour and shape, affects the fitness and evolution of flowering plant lineages (Moyroud and Glover, 2017). In flowers, size is determined by different factors: the number of organs in a whorl and the number of whorls per type (in particular, petals and stamens), as well as the overall size of each organ within a flower (Weiss *et al.*, 2005).

The genetic control of organ size has been largely studied in *Arabidopsis thaliana*, and gene networks controlling growth have been characterized in this species. In flowers, specifically in petals, the gene regulatory network includes transcription factors (TFs) and microRNAs that regulate developmental processes like primordium initiation and cell proliferation, expansion and differentiation (Huang and Irish, 2016). Among the genes involved in this process is *PETAL LOSS* (*PTL*), a TF that controls petal primordium initiation by regulating auxin accumulation; overexpression of *PTL* suppresses primordium growth and affects other developmental processes (Li *et al.*, 2008*a*; Lampugnani *et al.*, 2013). Other genes in the petal regulatory network identified as promoters of cell proliferation in the early phase of petal growth are *AINTEGUMENTA* (*ANT*) and *CYCD3;1*, which coordinate cell proliferation and lateral organ development (Mizukami and Fischer, 2000; Hu *et al.*, 2003); *ANGUSTIFOLIA3* (*AN3*) is involved in proliferation of epidermal and mesophyll cells in lateral organs like leaves and petals (Kawade *et al.*, 2013; Kinoshita *et al.*, 2022) while *AINTEGUMENTA-LIKE 6* (*AIL6*) has been reported as redundant to *ANT* and also as controlling flower development (Krizek, 2009). Genes involved in cell arrest, which is the moment when cells transit from a proliferative phase to a cellular expansion phase, are considered the major regulators that determine the final size and shape of an organ (Powell and Lenhard, 2012). *GROWTH-REGULATING FACTOR 9* (*GRF9*) acts as a negative regulator of leaf growth by restraining cell proliferation (Omidbakhshfard *et al.*, 2018). The *Arabidopsis grf9* mutants produce bigger leaves and strikingly also bigger petals, suggesting that some leaf growth regulators are also involved in petal development (Wang *et al.*, 2023); the ring-finger E3 ligase gene *BIG BROTHER* (*BB*) and the putative ubiquitin receptor gene *Defective in Abscission 1* (*DA1*) act synergistically to promote the transition from cell proliferation to expansion, restricting the proliferative phase (Disch *et al.*, 2006; Cattaneo and Hardtke, 2017).

Late phases of petal growth depend on cell expansion (Huang and Irish, 2016), which is regulated by ethylene (Pei *et al.*, 2013), and genes such as *BIG PETAL* (*BPEp*) *(*Szécsi *et al.*, 2006) and *NAC100*, which negatively control cell expansion (Szécsi *et al.*, 2006 ; Pei *et al.*, 2013).

Petal growth in roses showed that phytohormones and genes homologous to those found in *Arabidopsis* had a role in *Rosa chinensis* and *R. hybrida* petal development (Pei *et al.*, 2013; Han *et al.*, 2017). In spite of all the knowledge we have about petal growth in model systems, the dynamics of the regulatory mechanisms controlling organ size in non-model species remain elusive.

**Fig. 1.**
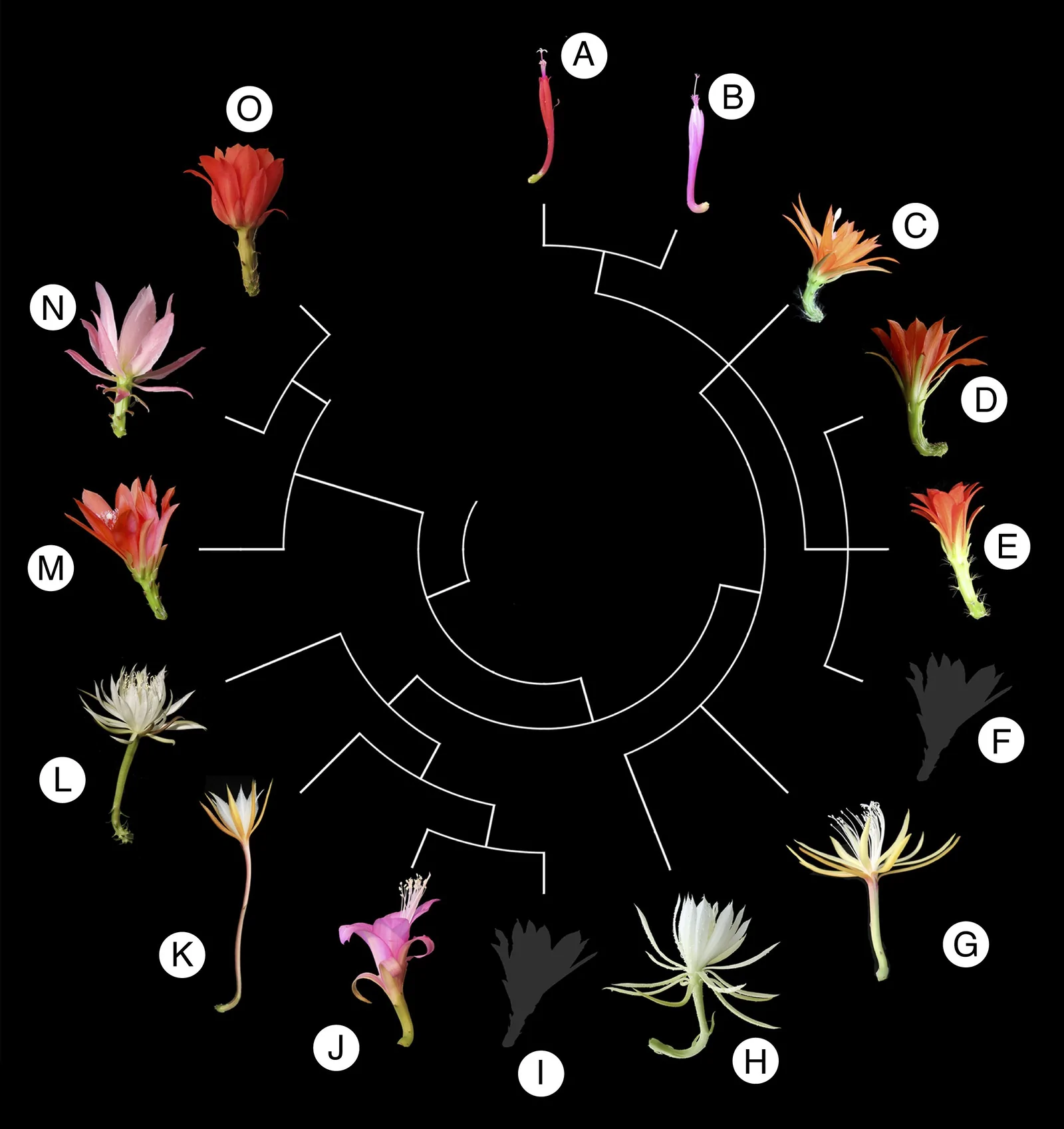
Simplified phylogeny of the *Disocactus* genus, representing the wide variety of flower morphologies (based on Cruz *et al.*, 2016). (A) *Disocactus eichlamii*, (B) *D. quezaltecus*, (C) *D. aurantiacus*, (D) *D. speciosus*, (E) *D. cinnabarinus*, (F) *D. bierianus* (no photographic record available), (G) *D. macranthus*, (H) *D. crenatus*, (I) *D. biformis* (no photographic record available), (J) *D. nelsonii*, (K) *D. anguliger*, (L) *D. lepidocarpus*, (M) *D. ackermannii*, (N) *D. phyllanthoides*, (O) *D. kimnachii*. Flowers are not to scale (photo credits: Salvador Arias).

**Fig. 2.**
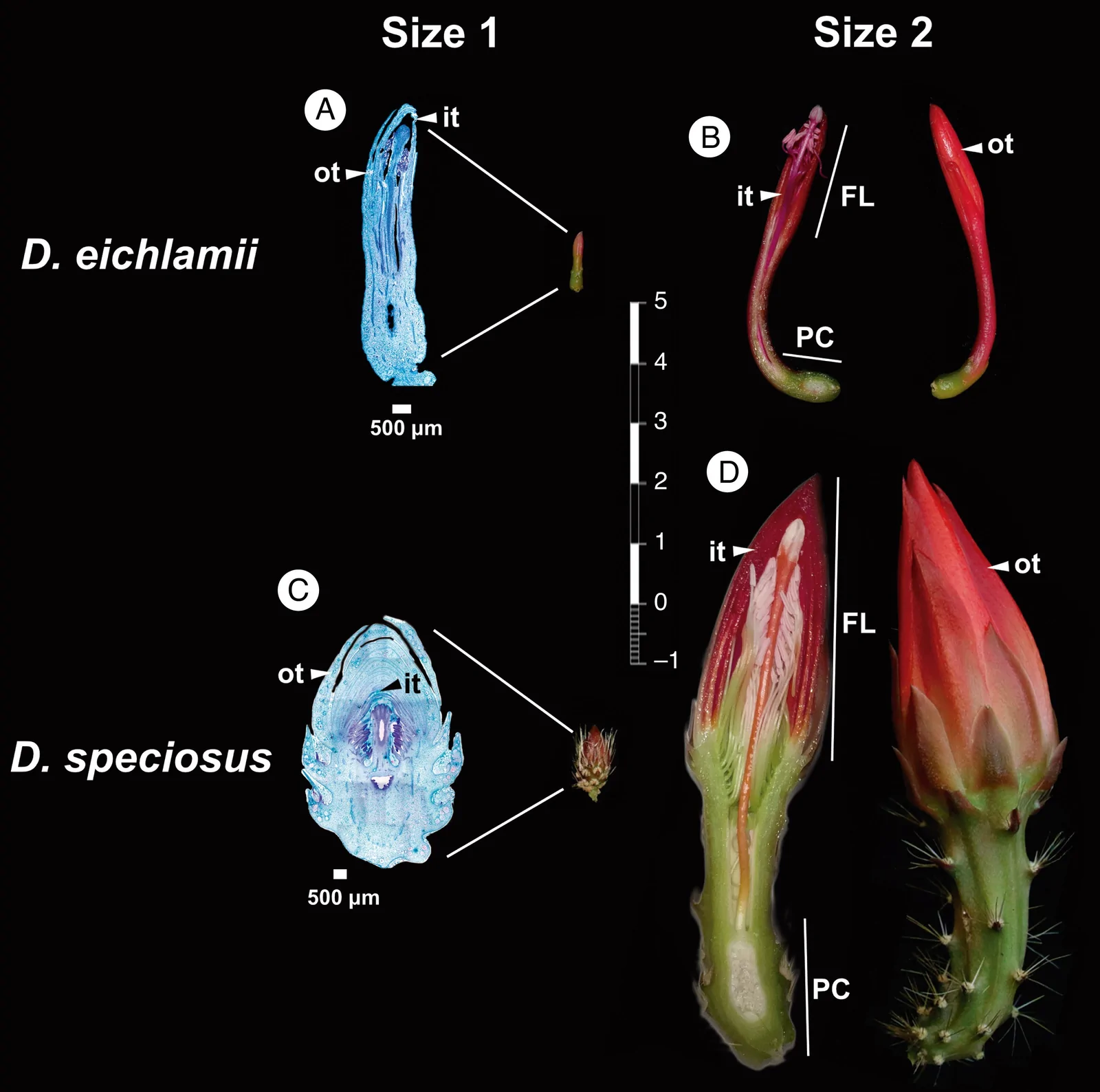
Flower buds of *D. eichlamii* and *D. speciosus*. Longitudinal anatomical sections of size 1 and longitudinal sections of fresh buds in size 2 (pre-anthesis). (A) *Disocactus eichlamii* flower bud 1 cm long (size 1) displaying pink tepals; the flower tube is under development and the pericarpel is green without spines and trichomes. A longitudinal view of an anatomical section of *D. eichlamii* flower bud size 1 (1 cm in length) is portrayed. Anatomical sections showed that all floral organs have been established, although different maturation stages can be observed. Scale bar = 500 μm. (B) *Disocactus eichlamii* flower buds in pre-anthesis (size 2), showing a reduced pericarpel and floral tube without areoles and spines and few magenta tepals. Scale bar is in cm. At this stage all organs have been completely developed. (C) *Disocactus speciosus* flower bud 1 cm long (size 1); no flower tube is visible and the brownish pericarpel is covered with spines and trichomes. An anatomical longitudinal section shows that all floral organs have been established and are in different maturation stages. Scale bar = 500 μm. (D) *Disocactus speciosus* flower bud in pre-anthesis (size 2), showing a large green pericarpel and floral tube covered by areoles, spines as well as numerous red tepals. Scale bar is in cm. A longitudinal section made by hand shows internal organization. it, innermost tepal; ot, outermost tepal; FL, perianth, stamens and stigma; PC, pericarpel and ovary.

Among angiosperms, Cactaceae species have undergone extreme developmental modifications of shoots and flowers, and probably no other plant family exceeds cacti in terms of structural diversity (Mauseth, 2006). Its members display a wide diversity in growth habit, including trees, bushes and geophytes (Vázquez-Sánchez *et al.*, 2012), as well as variation in shoot size (Mauseth, 2006). In this family, organ size variation is common not only in shoots (Mauseth, 2006) but also in other structures, such as leaves, spines, flowers and fruits. Furthermore, substantial organ size variation can be observed even among closely related species. For example, the genus *Disocactus*, which harbours 13 recognized species, encompasses taxa with contrasting flowers in terms of flower size, which range from 5 cm in length for *D. eichlamii* to 20 cm in *D. anguliger*. Colour is also diverse, with hues that range from white to red, while organ number in tepals and stamens is highly variable (Cruz *et al.*, 2016; Korotkova *et al.*, 2017). All these differences in floral features make *Disocactus* an interesting group for studying the genetic and ontogenetic differences underlying extreme morphological variation in flowers (Fig. 1). *Disocactus eichlamii* has a smaller flower, which reaches 5 cm length (Figs 1A and 2B), with 9–12 tepals, few stamens (∼20) and a stigma with five lobes. In contrast, *D. speciosus* has a large flower, reaching 10 cm length (Figs 1D and 2D), with 30–40 tepals, multiple stamens (∼200) and a stigma with ten lobes. Recent studies documenting early stages of flower development in these two species have shown that the contrasting final flower size and organ number could be related to size differences in the flower and ring meristems (Ramírez-Castro *et al.*, 2024). However, the genetic basis of flower size in general and the differential size between organs of these species in particular remain unknown. Here we integrate data from tepal cell measurements, cell counts and tepal histology and micromorphology of epidermis, as well as transcriptome analysis, to explore the developmental and genetic bases related to the contrasting flower size in *Disocactus eichlamii* and *D. speciosus* (Fig. 2).

## MATERIALS AND METHODS

### Plant material

We collected flower buds of the two species at two different sizes between January and March 2019 from 1200 to 1300 h. *Disocactus eichlamii* flower buds 1 cm in length (size 1) and 5 cm in length (size 2; pre-anthesis) were collected in the greenhouse of the Epiphytic Cacti Collection from the Botanical Garden of the Biology Institute at the Universidad Nacional Autónoma de México (UNAM), while *D. speciosus* Flower buds 1 cm in length (size 1) and 10 cm in length (size 2; pre-anthesis) were collected from wild specimens at the Reserva Ecológica del Pedregal de San Ángel (REPSA, UNAM). Hereinafter we refer to these size classes as size 1 and size 2 in both species (Fig. 2).

### Micromorphology of epidermis of tepals by scanning electron microscopy, and floral bud histology

The perigonium in Cactaceae develops in a spiral manner, with several series of tepals that display a morphological gradation from tepal–sepaloids (outermost) to tepal–petaloids (innermost). Based on this pattern, we collected the innermost and outermost tepals per size category (Fig. 2), dissecting five flowers of *D. speciosus* and five of *D. eichlamii*. In addition, we collected whole flower buds of size 1 from both species. Whole buds and the innermost and outermost tepals of the three size intervals for both species after collection were immediately fixed in FAA (ethanol, distilled water, formaldehyde and acetic acid in a ratio of 50:35:10:5) and put in a vacuum for 60 min. The FAA solution was removed after 48 h on the agitation table. The materials were washed with distilled water before processing.

The fixed tissues were dehydrated in a *tert*-butyl alcohol series (35–100 %, absolute), embedded in Paraplast (Leica) using a Paraplast infiltrator (Leica, HistoCore Arcadia H-C) at 60 °C and serially longitudinally sectioned at a thickness of 7 μm using a rotary microtome (American Optical 820). Subsequently, mounted slides were stained with safranin–fast green (Johansen, 1940). Slides were photographed (40× for *D. eichlamii* size 1; 10× for *D. eichlamii* size 2; 10× for *D. speciosus* size 1 and 2) under a bright-field microscope (Zeiss Axioscope) equipped with a digital camera (Panasonic imxCMOS), using the Rising View of AmScope software. Microphotographs were edited in Photoshop CS6.

For scanning electron microscopy micrographs, selected tepals were fixed in FAA following the method mentioned above. Tepal median cross-sections were dehydrated in an ethanol series (70–100 %), critical-point-dried with CO_2_ (Emitech K850), mounted on aluminium stubs and sputter-coated with gold (Quorum Q15OR). Images were taken at 500× magnification using a scanning electron microscope (SEM; Hitachi SU1510, 10 kV) with a digital camera attachment.

### Tepal cell count and measurement for statistical analysis

The statistical analysis of tepal epidermal cells was carried out using cleared tepals (chlorine + sodium hydroxide) for each position and floral size. Adaxial paradermal micrographs were taken at 40× magnification using a light microscope (Zeiss Axioscope) with a digital camera attached (Panasonic imxCMOS) and FlyCapture software. A total of 626 cells from the median zone, from five different tepals and different tepal positions (inner and outer tepals) for each size category used for the transcriptome analysis were measured in each species. The number of epidermal cells was determined using adaxial paradermal micrographs (10× magnification) of five tepals per position (inner, outer) from five different flower buds in pre-anthesis for both species (size 2; *n* = 25).

To estimate if there was a correlation between cell area and cell number with the final size of tepals, we measured five tepals from different flowers (inner and outer; Fig. 2) in size 2 floral buds (*n* = 25) from each species. Cell area, cell number and tepal longitude were calculated using Fiji v.2.9.0 (Schindelin *et al.*, 2012).

To evaluate differences between cell area, cell number and tepal length between species, sizes and tepal position (inner and outer), general linear models (GLMs) were adjusted. Analyses were made independently, using as predictor variables the interaction between species, stage and tepal position.

For the cell area and tepal length, a γ distribution with a logarithmic link function was used, appropriate for continuous and strictly positive data. For cell number, given its count nature, a Poisson distribution with a logarithmic link function was used. In both models, the significance of the effects was assessed using type III analysis of deviance, and *post hoc* multiple comparisons were performed using Tukey’s method, with correction for multiple comparisons using the Tukey HSD adjustment.

Comparisons of estimated means were obtained using the emmeans function from the R package of the same name, and significant contrasts were visualized by superimposed letters on the box plots. All analyses were performed in the R statistical environment (https://cran.r-project.org/).

### RNA extraction and RNA-seq library construction

Based on information regarding cactus flower ontogeny, two types of samples were dissected and processed separately: (1) samples completely derived from the floral meristem: stamens, stigma and tepals (together referred to as the floral (FL)), and (2) another composite section including vegetative tissue derived from a shoot apical meristem (SAM); the pericarpel and the enclosed inferior ovary (together referred to as the pericarpel (PC)). Floral tubes were excluded from the transcriptomic analysis since these structures are not homologous in the two species; in *D. eichlamii* the flower tube is developed by postgenital fusion of tepals (Buxbaum, 1953; Ramírez-Castro *et al.*, 2024), while in *D. speciosus* the tube is developed by intercalary growth of the pericarpel covering the floral tissue (inner tissue) while the outside comprises vegetative tissue (with areoles, spines and bracts). Six flowers of *D. eichlamii* (three biological replicates per size; Fig. 2A, B) and six of *D. speciosus* (three biological replicates per size; Fig. 2C, D) were collected (Supplementary Data Appendix S1). The dissected tissue was immediately flash-frozen in liquid nitrogen and stored at −80 °C until use.

Total RNA extraction was performed on 24 samples, 12 samples per species (6 from PC and 6 from FL) with the Spectrum™ Plant Total RNA Kit for Difficult Plant Tissues (Sigma‒Aldrich) following the manufacturer’s instructions. Purified RNA was quantified using a NanoDrop 2000 (Thermo Fisher Scientific, USA) and Qubit 4 (Thermo Fisher Scientific, USA) in RNA high-sensitivity mode. RNA integrity was assessed using a Bioanalyzer (Agilent) and visually inspected by electrophoresis in gel-chlorine (Aranda *et al.*, 2012). Library preparation and sequencing were performed by the Beijing Genomic Institute (BGI), Hong Kong, China, with the Illumina HiSeq™ 4000 platform with paired-end (PE) reads (2 × 150 nt); 20 million reads per sample were obtained, on average.

### Transcriptome *de novo* assembly and functional annotation

Raw reads were analysed with FastQC v.0.11.9 (Andrews and Krueger, 2010) and multiQC (Ewels *et al.*, 2016). Read pre-processing to remove adapters, duplicated sequences and low-quality reads was performed with Trimmomatic v.032 (Bolger *et al.*, 2014) using the following parameters: ILLUMINACLIP:TruSeq3-PE-2.PA:2:30:10 LEADING:25 TRAILING:25 MINLEN:75 SLIDING WINDOW:4:25.

The transcriptomes were assembled *de novo* for each species based on libraries from FL and PC, using Trinity 2.4.0 (Grabherr *et al.*, 2011) in paired-end mode (–paired-end). The reads were also aligned against the *Selenicereus undatus* genome (Zheng *et al.*, 2021) using Bowtie 2 (Langmead and Salzberg, 2012) through Galaxy (https://usegalaxy.org/). All reads unmapped to the *S. undatus* genome were reassembled using Trinity (Grabherr *et al.*, 2011). The *de novo* assemblies were filtered using cd-hit (Li and Godzik, 2006; Niu *et al.*, 2010) and a custom perl script to remove sequence duplicates.

The assembled transcripts were translated to their putative amino acid sequence by using Transdecoder through Galaxy (https://usegalaxy.org/) keeping only the longest ORF for subsequent transcript annotation. The transcriptome was annotated using PANNZER (Koskinen *et al.*, 2015; Törönen *et al.*, 2018) with the Viridiplantae database. Completeness of the assemblies was evaluated with BUSCO v.4 (Simão *et al.*, 2015) with the Viridiplantae, embryophyte and eudicot gene sets.

### Transcripts quantification, differential expression analysis and KEGG enrichment analysis

Transcript abundance was assessed by aligning the RNA-seq reads to the corresponding assembly and quantified with Salmon 1.5.2 (Patro *et al.*, 2017). Count matrices were created using the R package tximport (Soneson *et al.*, 2015). The counts were normalized using the trimmed mean of M values (TMM). Principal component analysis (PCA) was performed for each species using only floral data (FL, size 1 and size 2). Differential gene expression was analysed using the edgeR package (Robinson *et al.*, 2010; McCarthy *et al.*, 2012). The Benjamini–Hochberg procedure was used to adjust the resulting *P*-values to control for the false discovery rate (FDR). Any transcript with adjusted *P*-value of <0.05 and log fold change (FC) between the intervals FDR < 0.05 and log_2_FC > 1.5, FDR < 0.05 and log_2_FC < −1.5 was considered as differential expression. Using sequences of the differentially expressed genes (DEGs) we blasted against TAIR10 (Rhee *et al.*, 2003). BLAST results were used for the gene set enrichment analysis. Kyoto Encyclopedia of Genes and Genomes (KEGG) enrichment analysis of DEGs was performed through ShinyGO 0.80 (Ge *et al.*, 2020) using FDR cut-off <0.05. Selection of specific genes differentially expressed in the specific pathways was made based on enrichment results.

### Gene target selection and phylogenetic analysis

Direct selection of putative orthologues of genes previously reported as involved in growth processes, specifically those for the petal growth network (Huang and Irish, 2016; Han *et al.*, 2017; Ballerini *et al.*, 2019), were compared through BLAST using the BLASTN tool (Altschul *et al.*, 1990) against *Arabidopsis* genes. All BLAST results were filtered considering e-value, bit score and identity. To corroborate the orthology of the genes selected, gene trees were constructed using sequences downloaded from NCBI (https://www.ncbi.nlm.nih.gov/) and Phytozome v.13 (https://phytozome-next.jgi.doe.gov/), including species from Caryophyllales, rosids and asterids, as well as species from the ANITA clade. Multiple sequence alignments were made using MAFFT 0.7 (Kuraku *et al.*, 2013; Katoh *et al.*, 2019). Maximum likelihood phylogenetic analyses were performed using IQ-TREE 11.6.12 with a total of 1000 bootstrap replicates to assess the statistical support of the resulting trees. Tree visualization and editing was done with FigTree v.1.4.4 (http://tree.bio.ed.ac.uk/software/figtree/).

## RESULTS

### Morphological and anatomical nature of differences in flower size

To explore morphological differences during flower growth, we performed anatomical sections on flower buds from size 1 and by hand longitudinal sections of fresh buds from size 2 (pre-anthesis). These sections showed that, in both species, the 1 cm long (size 1) floral bud had well-differentiated floral organs (Fig. 2A, C).

**Fig. 3.**
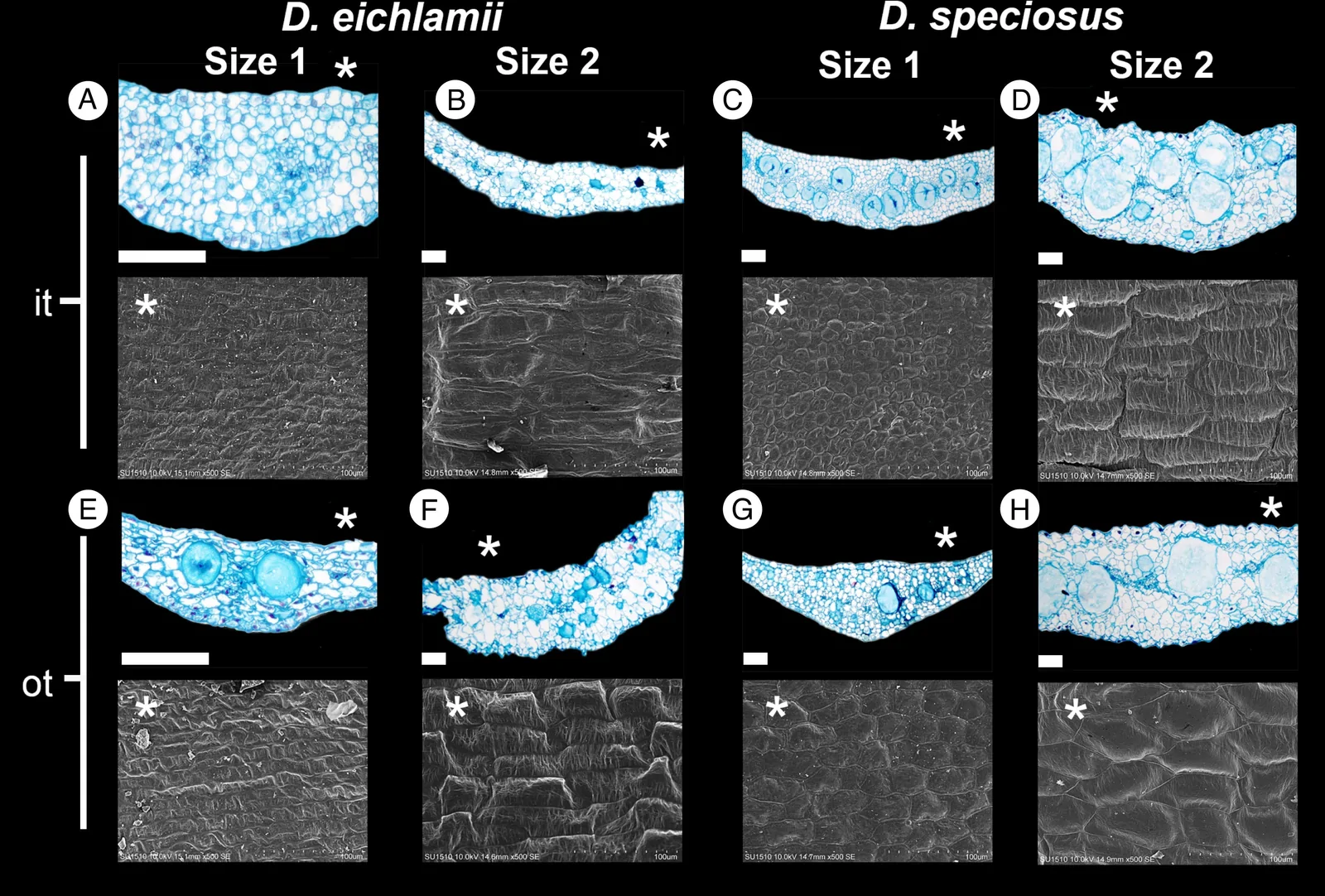
Median sections of the innermost (it) and the outermost (ot) tepals in *D. eichlamii* and *D. speciosus*, and a polar view of adaxial cell micrographs. (A) *Disocactus eichlamii*, size 1, innermost median tepal section at 40× magnification. Below is a polar view of adaxial cells in an SEM micrograph at 500× magnification. (B) *Disocactus eichlamii*, size 2, innermost median tepal sections at 10× magnification. Below is a polar view of adaxial cells in an SEM micrograph at 500× magnification. (C) *Disocactus speciosus*, size 1, innermost median tepal sections at 10× magnification. Below is a polar view of adaxial cells in an SEM micrograph at 500× magnification. (D) *Disocactus speciosus*, size 2, innermost median tepal section at 10× magnification; parenchyma cells are bigger than those observed in parenchyma cells in *D. eichlamii* in the same size/category. Large cells with polysaccharides are also observed. (E) *Disocactus eichlamii*, size 1, outermost median tepal section at 40× magnification. Below is a polar view of adaxial cell in an SEM micrograph at 500× magnification. (F) *Disocactus eichlamii*, size 2, outermost median tepal section at 10× magnification. Below is a polar view of adaxial cells in an SEM micrograph at 500× magnification. (G) *Disocactus speciosus*, size 1, outermost median tepal section at 10× magnification. Below is a polar view of adaxial cells in an SEM micrograph at 500× magnification. (H) *Disocactus speciosus*, size 2, outermost median tepal section at 10× magnification. Below is a polar view of adaxial cells in an SEM micrograph at 500× magnification. Scale bar = 100 μm; *, adaxial face.

Across the analysed tepal sections in size 1 and size 2, we observed notable differences in cell sizes, suggesting the possibility that cells of size 1 are still under active cell proliferation in both the inner and outer tepals of both species (Fig. 3A, C, E, G), while in size 2 buds (pre-anthesis) tepal cells are longer as a result of cell expansion and elongation (Fig. 3B, D, F, H).

Furthermore, SEM micrographs of tepal epidermis showed that the area and shape of epidermal cells in buds of size 1 in both species are comparable (Fig. 3A, C, E, G); however, SEM photographs from size 2 tepals showed that cells in *D. eichlamii* are more elongated and narrower (Fig. 3B, F) than those observed in size 2 in *D. speciosus* (Fig. 3D, H), where cells are more rounded and smaller. The difference is more evident in inner tepal cells (Fig. 3B, D).

### The developmental nature of tepal size variation in *Disocactus*

To establish if there is a correlation between cell size and overall flower size in the two *Disocactus* species, cell count and cell area measurements were performed on the innermost and outermost tepals in the pre-anthetic flowers (Fig. 3B, D, F, H).

**Fig. 4.**
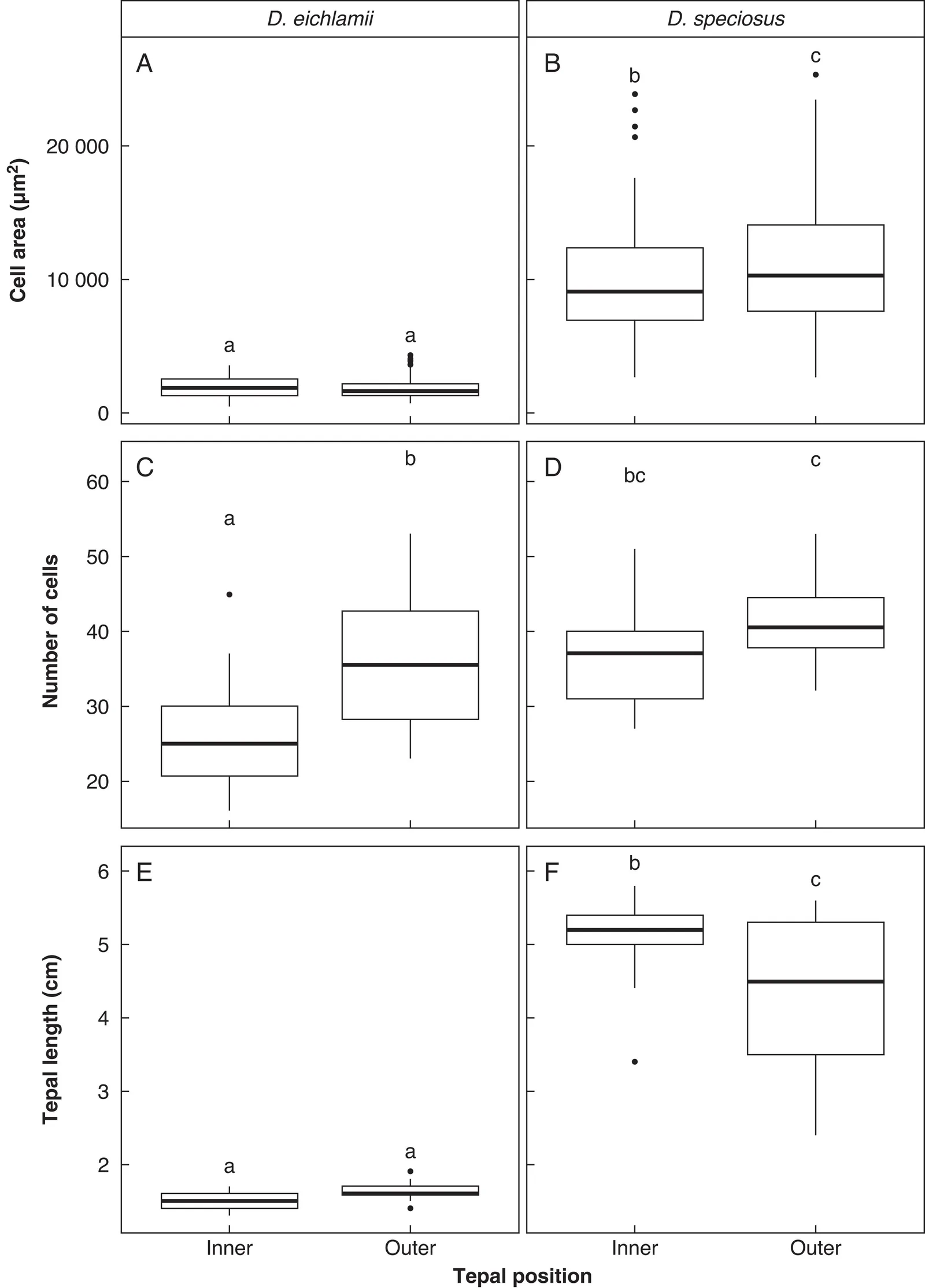
Cell area, cell number and tepal length in D. eichlamii and D. speciosus. (A, B) Estimation of epidermal cell area (μm2) in outer and inner tepals of pre-anthetic flower buds (size 2). (A) *Disocactus eichlamii*. (B) *Disocactus speciosus*. Boxes represent median and interquartile ranges. Different letters above boxes within the same developmental stage indicate statistically significant differences at the 0.05 level based on Tukey’s *post hoc* test. (C, D) Estimation of epidermal cell number per 10 μm^2^ in inner and outer tepals from pre-anthesis buds (size 2). (C) *Disocactus eichlamii*. (D) *Disocactus speciosus*. Significant differences in cell number between tepal positions were only observed in *D. eichlamii*. When comparing species, *D. speciosus* shows significantly more cells than *D. eichlamii*. Different letters indicate significant differences at the 0.05 level based on a Poisson model with Tukey adjustment. (E, F) Longitudinal tepal length (cm) in inner and outer tepals from pre-anthesis flower buds (size 2). (E) *Disocactus eichlamii*. (F) *Disocactus speciosus*. Tepals in *D. speciosus* are significantly longer than those in *D. eichlamii*, with inner and outer positions showing differences in length only in *D. speciosus*.

Our statistical analyses showed that cell size, cell number and tepal length in *Disocactus* are determined by a specific pattern dependent on species and the position of the tepals within the perigonium (Supplementary Data Appendix S2). A GLM with γ distribution and log link revealed that cell area is statistically different among species (*β* = 1.639, s.e. = 0.063, *t* = 25.98, *P* < 0.001); this difference is even more evident when considering also tepal position (*β* = 0.173, s.e. = 0.077, *t* = 2.26, *P* = 0.024), whereas the general effect of tepal position was not significant (*β* = −0.043, s.e. = 0.066, *t* = −0.66, *P* = 0.510). This indicates that the influence of tepal position on cell area depends on the species being analysed (Fig. 4A, B). *Post hoc* comparisons with Tukey correction showed that *D. speciosus* exhibits significantly larger cell areas than *D. eichlamii* in both outer tepals (estimate = −1.6391 μm^2^, *P* < 0.0001) and inner tepals (estimate = −1.8125 μm^2^, *P* < 0.0001). In *D. speciosus*, inner tepals have slightly smaller cell areas than outer ones (estimate = −0.130 μm^2^, *P* = 0.0059), whereas in *D. eichlamii* no significant differences were found between tepal positions (estimate = 0.0433 μm^2^, *P* = 0.9125). These results indicate that *D. speciosus* has significantly larger cells than *D. eichlamii* in both tepal positions and that in *D. speciosus* cell area varies with tepal position, whereas in *D. eichlamii* tepal position has no significant effect.

To evaluate the contribution of cell expansion to these phenotypes, we counted epidermal cells in the median region of tepals from pre-anthesis floral buds (Fig. 4C, D). A GLM with Poisson distribution showed that cell number depends significantly on species (*β* = 0.375, s.e. = 0.052, *z* = 7.21, *P* < 0.001), tepal position (*β* = 0.327, s.e. = 0.052, *z* = 6.26, *P* < 0.001) and their interaction (*β* = −0.217, s.e. = 0.069, *z* = −3.13, *P* = 0.002). Overall, *D. speciosus* had more cells per unit area than *D. eichlamii*, although the distribution pattern differed between species. In *D. eichlamii*, inner tepals had significantly fewer cells than outer tepals (*P* < 0.0001), whereas in *D. speciosus* no significant difference was found between tepal positions (*P* = 0.0772). When comparing inner tepals across species, *D. speciosus* showed significantly more cells than *D. eichlamii* (*P* = 0.0030).

To assess whether these cellular differences translated into morphological variation, we analysed tepal length using a GLM with γ distribution and log link (Fig. 4E, F). The model indicated significant main effects of species (*β* = 1.236, s.e. = 0.041, *t* = 30.45, *P* < 0.001) and tepal position (*β* = 0.094, s.e. = 0.041, *t* = 2.33, *P* = 0.022), as well as a significant interaction between species and position (*β* = −0.281, s.e. = 0.057, *t* = −4.89, *P* < 0.001). *Post hoc* comparisons showed that *D. speciosus* had significantly longer outer tepals than *D. eichlamii* (estimate = −0.9549 cm, *P* < 0.0001) and also exhibited significant size differences between inner and outer tepals (estimate = 0.1865 cm, *P* = 0.0001). In contrast, *D. eichlamii* showed no significant difference in size between tepal positions (estimate = −0.0943 cm, *P* = 0.0996). Moreover, inner tepals of *D. speciosus* were significantly longer than those of *D. eichlamii* (estimate = −1.2358 cm, *P* < 0.0001).

Altogether, our results demonstrate a direct relationship between cell area, cell number and final organ length. Flowers of *D. eichlamii*, which are ∼5 cm long, exhibit smaller and fewer cells, resulting in shorter tepals. In contrast, *D. speciosus* produces flowers up to 10 cm long, with larger and more numerous cells, yielding longer tepals. These differences suggest that divergent mechanisms of cellular growth regulation have contributed to the evolution of floral size in *Disocactus*.

### Transcriptomic profiles for *Disocactus* species are similar in sizes 1 and 2

To explore transcriptional differences between *D. eichlamii* and *D. speciosus*, we sequenced and assembled the transcriptome of both species of size 1 and 2. The number of transcripts for *D. speciosus* and *D. eichlamii* was 55 464 and 53 931, respectively, including isoforms per gene (Table 1). Both transcriptomes have an average completeness of ∼90 % as inferred through comparison with Viridiplantae, Embryophyte and the Eudicot BUSCO gene sets (Supplementary Data Appendix S3).

**Table 1.** *De novo* transcriptome assembly results.

	*Disocactus speciosus* (transcripts)	*Disocactus eichlamii* (transcripts)
Number of sequences	99 167	102 259
Total length (nt)	131 595 696	135 694 494
Mean length (nt)	1327	1327
N50 (nt)	1953	1872
GC (%)	41.43	43.07

Of all transcripts of *D. speciosus* (28 381) and *D. eichlamii* (25 191), 51.17 and 46.70 %, respectively, were annotated, including some isoforms. A comparative analysis using the genome of *S. undatus* showed that 15 436 transcripts in *D. speciosus* and 15 903 transcripts in *D. eichlamii* are putative orthologues of *S. undatus* genes.

**Fig. 5.**
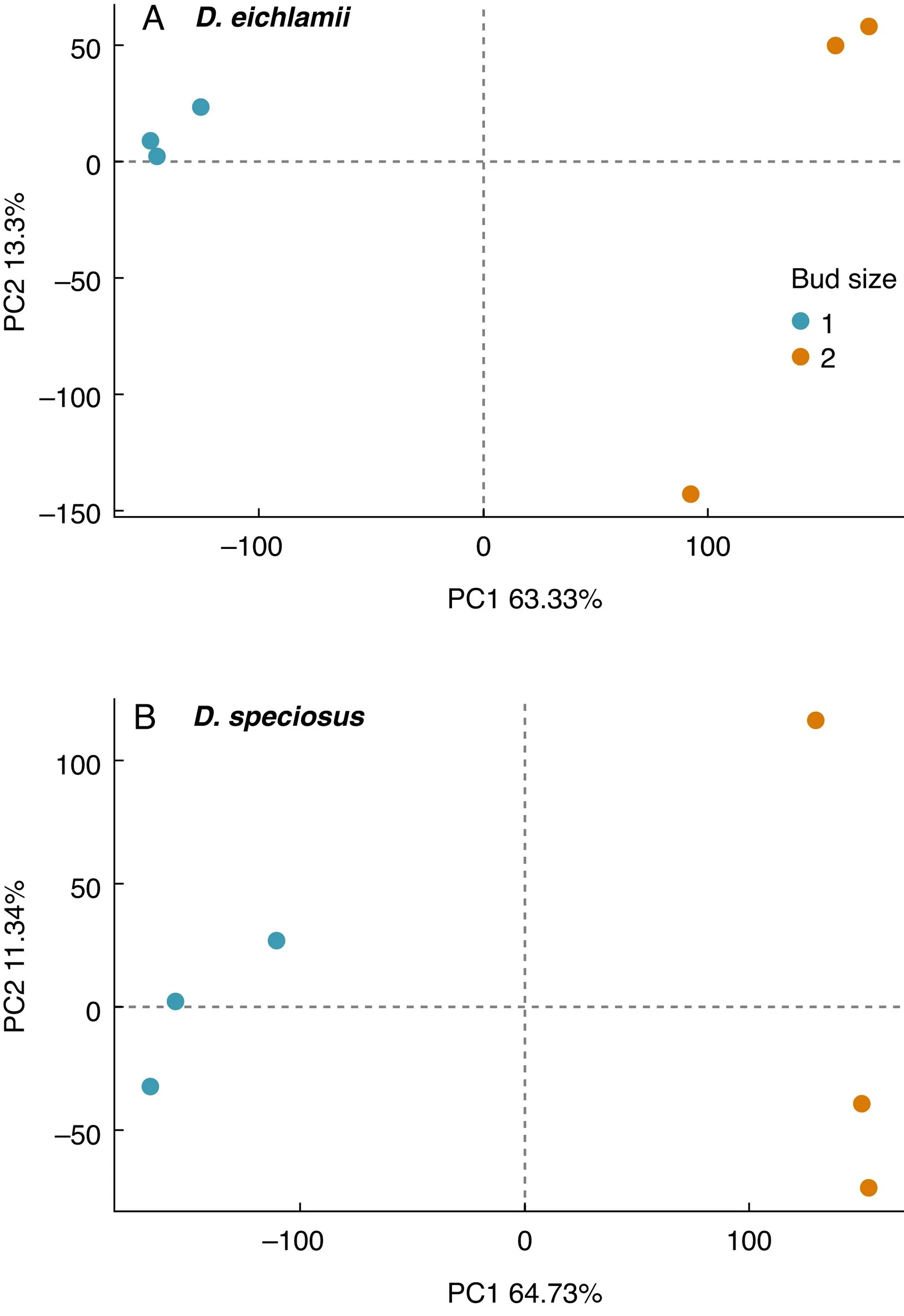
PCA based on TMM values from three biological replicates of each size category in (A) *Disocactus eichlamii* and (B) *D. speciosus*. The PCA shows that flower buds in size 1 and size 2 have similar transcriptional landscapes.

Transcriptome sizes and completeness were comparable between species, which was confirmed by a PCA performed on the three biological replicates of each size category per species, showing that the two first principal components (PCs) explained >75 % of the variance, with PC1 accounting for 64.73 and 63.33 % and PC2 accounting for 11.34 and 13.3 % in *D. speciosus* and *D. eichlamii*, respectively. The PCA (Fig. 5, Supplementary Data Appendix S4) for both species shows that the floral tissue (FL) transcriptomes for sizes 1 and 2 are close to each other, suggesting that the magnitude of the main trends of variation of the transcriptional landscape of these tissues in the two species at these developmental stages are similar.

### Identification of genes and pathways associated with growth in size

Our statistical analysis of epidermal cells suggests that differences in final overall size are related to differences in cell proliferation rates and cell elongation as well as cell expansion patterns (Fig. 4). We thus surveyed our transcriptomes for genes involved in cell cycle regulation and organ growth.

**Fig. 6.**
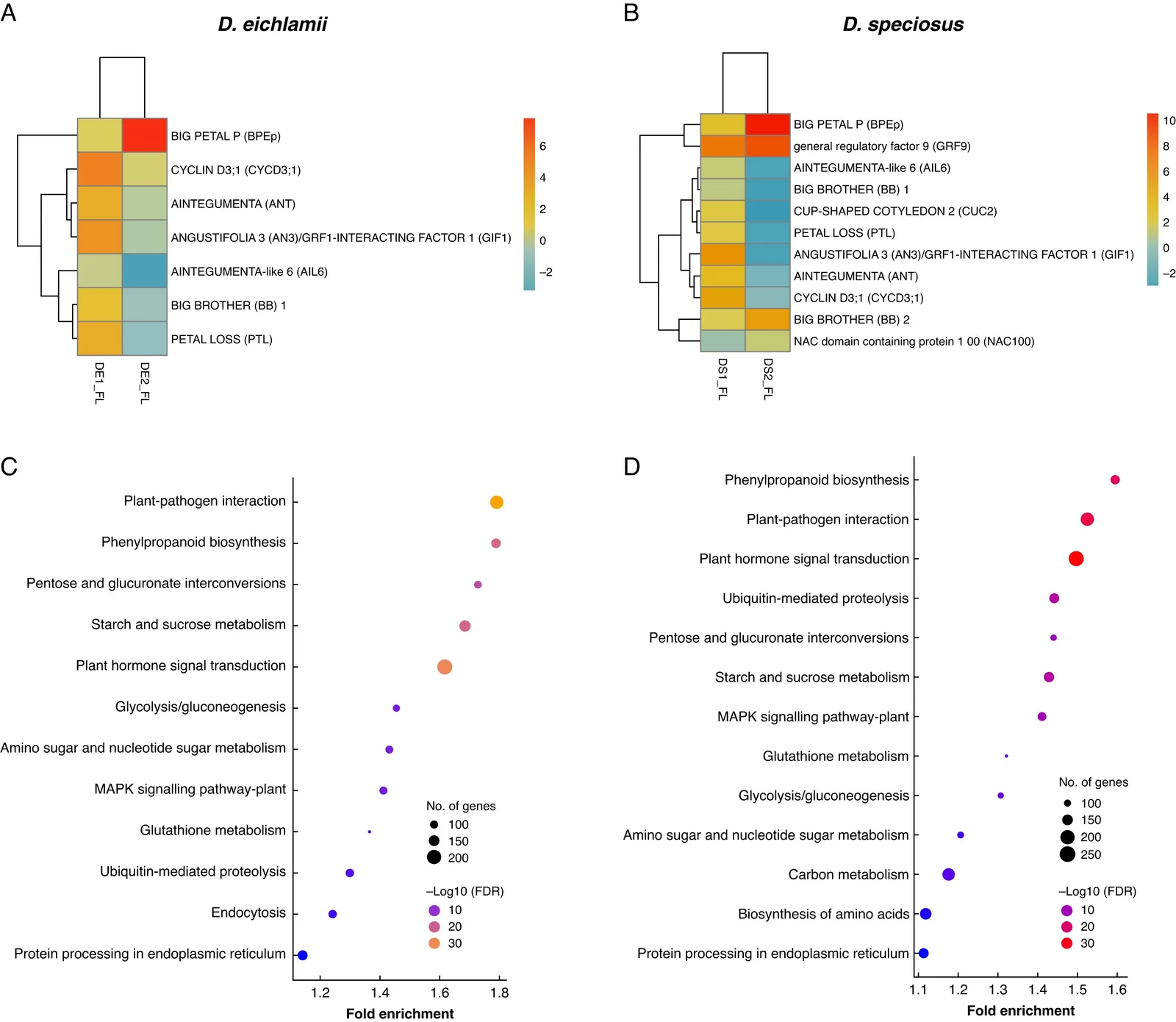
Differentially expressed genes in the tepal growth pathways between size 1 and 2 buds and the most significant GO pathways, based on the enrichment test. (A) *Disocactus eichlamii*, DE1_FL vs DE2_FL. Seven orthologues of genes previously reported in the petal growth pathway showed differential expression in this species. In this case, only one copy of the *DeiBB* orthologue was differentially expressed. (B) *Disocactus speciosus*, DS1_FL vs DS2_FL. Eleven orthologous genes in the petal growth pathway were differentially expressed in this species, including two copies of the *DspBB* orthologous gene. (C, D) KEGG enrichment of 15 pathways in (C) *D. eichlamii* and (D) *D. speciosus* for genes differentially expressed in flower tissue between growth stages.

In *D. eichlamii*, we found putative orthologous genes related to petal growth that were differentially expressed (*P* < 0.05, FDR < 0.05 and log_2_FC > 1.5) between bud sizes 1 and 2. For instance, *PETAL LOSS* (*DeiPTL*) and the putative orthologues of *AINTEGUMENTA* (*DeiANT*) and *AINTEGUMENTA-like 6* (*DeiAIL6*) were upregulated in size 1 vs size 2 buds. These genes act redundantly and control growth during the proliferative phase. Another upregulated TF with a role in cell proliferation that was differentially expressed in size 1 vs size 2 buds was *ANGUSTIFOLIA 3* (*AN3*)*/GRF1-INTERACTING FACTOR 1* (*DeiGIF1*). Furthermore, three isoforms of *CYCLIN D3;1* (*DeiCYCD3;1*), which has been documented to coordinate cell proliferation, were equally upregulated in size 1 vs size 2 (Fig. 6A).

Interestingly, we found two copies of the putative orthologue of *BIG BROTHER* (confirmed through phylogenetic trees; Supplementary Data Appendix S5), an E3 ubiquitin ligase-encoding gene involved in limiting organ size through the control of cell proliferation. In our data, *DeiBB1* with two isoforms was upregulated in size 1 vs 2 flowers, while *DeiBB2* expression was not significant, nor different between sizes. Regarding genes involved in the control of cellular expansion, we found three isoforms of the putative orthologue of *BIG PETALp* (*DeiBPp*), a basic helix-loop-helix TF, that restricts petal size by controlling cell expansion. These transcripts were downregulated in size 1 compared with size 2 buds.

Notably, even though mature flowers of *D. speciosus* are twice the size of flowers from *D. eichlamii*, the gene expression patterns in *D. speciosus* were similar for the genes described above, with the exception of *CUP-SHAPED COTYLEDON* 2 (*DspCUC2*), a gene with a role in limiting cell divisions and organ boundaries, which was upregulated in size 1 vs 2, and only detected in *D. speciosus* (Fig. 6B). In contrast, genes involved in limiting cell size, such as *GROWTH-REGULATING FACTOR 9* (*DspGRF9*) and an *NAC* domain containing protein 100 (*DspNAC100*) were both only significantly downregulated in *D. speciosus.* It is worth mentioning that while in *D. eichlamii* three isoforms of the putative orthologue of *DeiBPp* were found, in *D. speciosus* we identified two isoforms of the putative orthologue *DspBPp* gene that were also downregulated in size 1 vs 2 (Supplementary Data Appendix S6). On the other hand, in *D. eichlamii* we also identified two copies of the orthologous gene of *BB*; while in *D. speciosus DspBB1* was upregulated, the same copy in *D. eichlamii* (*DeiBB1*) was not. Furthermore, while in *D. speciosus DspBB2* with two isoforms was downregulated, in *D. eichlamii DeiBB2* did not show significant expression.

To investigate the major pathways associated with DEGs, we performed a gene set enrichment analysis. The KEGG enrichment showed that several processes were enriched in both species in a similar magnitude: plant hormonal signalling transduction had a fold enrichment of 1.6 in *D. eichlamii* vs 1.5-fold enrichment in *D. speciosus*; plant pathogen interaction and phenylpropanoid biosynthesis were also enriched in similar magnitudes (Fig. 6C, D). Notably, many DEGs were associated with plant hormonal signalling transduction pathways, including *AUXIN TRANSPORTER PROTEIN 1* (*AUX1*), *TRANSPORT INHIBITOR RESPONSE* 1 (*TIR1*), *CYTOKININ RESPONSE 1* (*CRE1*), *GRETCHEN HAGEN 3* (*GH3*), *BRIDGING INTEGRATOR 2* (*BRI2*) and *CYCLIN D3* (*CYCD3*) related to plant growth, cell division and cell elongation, and stem growth, among other developmental processes (Supplementary Data Appendix S7).

Further exploring DEGs in this pathway, we found 179 transcripts shared between species, while 72 are only differentially expressed in *D. speciosus* but not in *D. eichlamii*; in contrast, 10 are exclusively differentially expressed in *D. eichlamii* (Supplementary Data Appendix S8), suggesting that although this pathway is enriched in the two species, transcriptional regulation is different and could be linked to the different organ size observed in pre-anthetic flowers. Genes of the families *YUCCA* (*YUC*), *PIN-Like* (*PIL*) and *ARABIDOPSIS RESPONSE REGULATOR* (*ARR*) are among those only differentially expressed in *D. speciosus*, while genes of the families *TOLL/INTERLEUKIN-1 RECEPTOR* (*TIR*), *TGACG MOTIF-BINDING FACTOR* (*TGA*) and *POLY(A)-NUCLEASE* (*PAN*) are differentially expressed only in *D. eichlamii*.

## DISCUSSION

Despite efforts to understand the genetic control of organ size in plants, and the knowledge gathered from studies in *Arabidopsis* and other model organisms, we still lack a comprehensive understanding about growth dynamics and the genetic bases that modulate the size of specific organs in most angiosperms, especially perennials with long life cycles, like Cactaceae. In plants, the final dimensions of vegetative structures such as leaves, roots and stems can vary significantly depending on environmental factors such as the amount of light, temperature variations or soil conditions. In contrast, flower organ size is remarkably constant within a given species, indicative of a strong endogenous control during morphogenesis (Varaud *et al.*, 2011). Plant organ growth occurs by an initial proliferative phase in which cell numbers increase while their size remains fairly constant, followed by a dramatic cell size increase that ceases when the final size of the organ is reached (Li *et al.*, 2008*b*). Here we studied two cactus species to understand the genetic bases underlying contrasting flower size in two closely related species, leveraging cellular data as well as transcriptomic data from different flower bud stages of *D. speciosus* and *D. eichlamii* (Fig. 2). In the case of the two species analysed here, our cell measurements and cell counts suggest that differential cell proliferation and expansion dynamics underlie the contrasting size observed in pre-anthetic flowers, which was quantitatively assessed through the analysis of epidermal cells in tepals from two different sections or positions (termed inner and outer) within the flower bud (Fig. 4). *Disocactus speciosus* has larger tepals with bigger cells, which also correlates with the final size of the overall flower (10 cm long), while *D. eichlamii* has smaller cells, consistent with final tepal size and final overall flower size (5 cm long; Fig. 4A, B). Interestingly, cell numbers at size 2 are not significantly different between species, contrary to initial expectations, as final tepal size in *D. speciosus* is more than twice that of tepals in *D. eichlamii* (Fig. 4E, F). This suggests that final floral organ size could be more closely associated with cell elongation and expansion than with proliferation. It is worth mentioning that cells in size 2 tepals from *D. eichlamii* are longer than those in *D. speciosus*, which are more rounded in the same stage. This could be associated with anisotropic growth of cells in *D. eichlamii* (Fig. 3B, inner tepals). However, in terms of cell area, cells in *D. speciosus* are bigger than cells in *D. eichlamii* (Fig. 4B). These results suggest that the two species differ in the molecular mechanisms regulating tepal size: in *D. eichlamii*, cell area remains constant between positions while cell number varies, indicating a primarily proliferative control. In contrast, *D. speciosus* maintains a constant number of cells between positions, while cell size varies, suggesting control through cell expansion. Thus, *D. speciosus* combines enhanced cell proliferation and expansion to achieve a larger final tepal size.

Given these morphological and anatomical observations, we expected to find different gene expression patterns for transcripts related to cell expansion. However, we found that overall gene expression patterns are very similar in the two species (Fig. 5). Our targeted search for genes involved in regulating the cellular mechanisms underlying organ growth – cell proliferation and cell expansion (Huang and Irish, 2016; Han *et al.*, 2017; Ballerini *et al.*, 2019) – detected 11 DEGs in *D. speciosus* and 7 DEGs in *D. eichlamii* (*P* < 0.05, FDR <0.05 and log_2_FC > 1.5). For the genes shared between species, four are characterized as being involved in the promotion of organ growth by maintaining cell proliferation potential: *ANT*, *GIF1*, *AIL6* and *PTL*, which in both species showed similar expression patterns (upregulated in size 1 vs 2). Another gene that was differentially expressed in equivalent stages of each species was *BIG BROTHER*, a gene related to the repression of plant organ growth through the promotion of the transition from cell proliferation to cell expansion, limiting the proliferative phase (Disch *et al.*, 2006). Thus, this is an independent line of evidence that suggests that final bud size in *D. speciosus* is influenced by a longer period of cell elongation and expansion rather than an increase in cell proliferation (Figs 4 and 6). *BIG BROTHER* is expressed in all plant tissues; however, loss-of-function mutants in *Arabidopsis* seem to be mostly affected in petal and stem growth (Li *et al.*, 2008*b*). While we found two copies of *BB* in each species, only in *D. speciosus* were the two genes differentially expressed, in contrast with *D. eichlamii*, where only one of these copies was differentially expressed (Fig. 6A). These two putative copies (Supplementary Data Appendix S5) can be the product of gene duplication, which could have reduced selective constraint and thus enable the acquisition of novel gene functions, as has been suggested for other plants (Panchy *et al.*, 2016). Here, the copy *DspBB*1 is homologous to *BB* from *Arabidopsis* and could also act to restrict cell proliferation (Disch *et al.*, 2006). The function of the second copy, *DspBB2*, is still unknown but we found it is also present in other Cactaceae and several Caryophyllales (Supplementary Data Appendix S5). It has been reported that small changes in the level of expression of *AtBB* can substantially alter final organ size, suggesting a central regulatory role for this gene in growth control, as different levels of *BB* overexpression lead to moderate to strong reductions in petal and sepal size, stem thickness and flower biomass accumulation (Disch *et al.*, 2006). Although in both species analysed here *BB1* is upregulated in stage 1, its expression is slightly higher in *D. speciosus* compared with the expression of the orthologous copy in *D. eichlamii* (Fig. 6A, B). In contrast, *DspBB*2 is downregulated in size 2 of *D. speciosus* (Fig. 6B). These observations suggest that future tissue- and organ-specific analyses could help unravel the role of *BB* genes in modulating organ size in the *Disocactus* species studied here.

While in Cactaceae polyploidy is present in several genera, like *Opuntia*, *Consolea*, *Echinocereus* and *Mammillaria* (Negrón-Ortiz, 2007), we do not know if a whole-genome duplication (WGD) in *Disocactus* exists. Thus, either WGD or other genomic mechanisms could explain *BB* duplication, such as subgenomic duplication events like tandem duplications, duplication mediated by transposable elements (TEs), segmental duplications or retroduplications (reviewed by Panchy *et al.*, 2016). The fact that *BB* is duplicated in the surveyed Cactaceae is very interesting since size alterations in this family can be extreme, with various species that display gigantism or dwarfism in vegetative and reproductive structures; thus future research on the role of *BB* on extreme phenotypes in different cactus lineages should be pursued. The ubiquity of gene paralogues in Cactaceae is unknown, since just a few genomes are available for species within this family (Copetti *et al.*, 2017; Amaral *et al.*, 2021; Zheng *et al.*, 2021), which suggests that comparative genomics could be another important research venue in Cactaceae as a means to further understand the genomic evolutionary process underlying contrasting plant sizes among members of this group of plants.

Additionally, we documented the expression of genes involved in the negative control of cell expansion in both species. *BIG PETALp*, a gene that limits petal size by restricting postmitotic cell expansion (Szécsi *et al.*, 2006; Varaud *et al.*, 2011), was differentially expressed in *Disocactus*. In the perigonium, *D. speciosus* develops bigger cells compared with those observed in *D. eichlamii* in both outer and inner tepals (Figs 3 and 4). While the initial (stage 1) expression of *BPp* is quite similar between species, by stage 2 *D. eichlamii* has lower downregulation when compared with *D. speciosus* (Fig. 6A, B). Small differences in expression make it difficult for us to develop a hypothesis of this gene’s role in cell expansion, as we could expect that if both species have a similar expression of *BPp*, their cell size should be similar, but in *D. eichlamii* cells are longer and narrower when compared with *D. speciosus*, which could be a result of anisotropic elongation or other unknown gene-regulatory interactions (Fig. 3).

Additionally, we found three genes differentially expressed only in *D. speciosus*: *CUP-SHAPED COTYLEDON 2* (*CUC2*), *GROWTH-REGULATING FACTOR 9* (*GRF9*) and *NAC100.* The first two transcripts are related to the proliferative phase, while the third has been associated with the control of cell expansion. Thus, these genes could explain why, in spite of having very similar expression patterns, our two species develop substantial differences in flower size. *NAC100* is a TF from the NAM subfamily of the NAC-domain TF family that is induced by ethylene, a hormone associated with organ maturation (Pei *et al.*, 2013). The overexpression of *NAC100* represses cell expansion (Pei *et al.*, 2013), controlling petal growth. It is possible that this TF is producing larger cells in *D. speciosus* during flower bud growth, given the particular gene-regulatory interactions that favour cellular expansion reported before, as the overexpression of *RhNAC100* in *Arabidopsis* substantially reduced petal size by repressing cell expansion, while silencing *RhNAC100* in rose petals using virus-induced gene silencing significantly increased petal size and promoted cell expansion in the petal abaxial subepidermis, with an increase in cell area and a decrease in relative cell density when compared with the wild-type (Pei *et al.*, 2013). This is consistent with our cellular observations, as tepals of *D. speciosus* showed an increase in cell area while having a similar cell density to *D. eichlamii* (Fig. 4), suggesting that the *DsNAC100* putative orthologue, which increases its expression in size 2 buds of *D. speciosus*, could act in a way similar to that reported for *R. chinensis*; however, further experiments involving qRT–PCR, gene silencing approaches etc. should be performed to test this hypothesis.

Enrichment analysis showed that transcripts for the KEGG category ‘Plant hormone signal transduction’ were enriched in both species (Fig. 6C, D). Further analyses of genes involved in this biological process showed additional differences in expression between species, as 179 DEGs were shared between species, but 72 were differentially expressed only in *D. speciosus*, while 10 transcripts were exclusively differentially expressed in *D. eichlamii* (Supplementary Data Appendix S8). These DEGs regulate the biosynthesis and metabolism of six plant hormones: abscisic acid, gibberellin, auxin, jasmonate, cytokinin and ethylene. DEGs associated with auxins were the most ubiquitous, suggesting that auxins played a role in regulating flower bud development. As ethylene is involved in organ maturation, we analysed gene targets of this hormone and found that *ETHYLENE RECEPTOR* (*ETR*), *ETHYLENE-INSENSITIVE 3* (*EIN3*) and *EIN3-BIDING F-BOX* (*EBF*) were differentially expressed in *D. speciosus* but not in *D. eichlamii* (Supplementary Data Appendix S8). At the cellular level, cell expansion-related processes, such as cell wall synthesis, cell turgidity and cytoskeleton remodelling, are significantly affected by ethylene treatment (Pei *et al.*, 2013), which indicates that the differential expression of some of the targets of this hormone could underlie differential organ growth. Nevertheless, the integration of hormonal signal transduction pathways in this species and their role in the growth process of the different organs of its flower needs further investigation and could help elucidate the role that contrasting hormone expression has on final organ size in diverse species of Cactaceae. Another explanation for the contrasting sizes of floral organs in our species could be related to endoreduplication, which might be taking place in tepals of *D. speciosus*, as this phenomenon is linked to significantly larger cells in leaves and sepals of *Arabidopsis* and petals of cabbage (De Veylder *et al.*, 2002; Kudo and Kimura, 2002), as well as larger root cells and endosperm in maize. During endoreduplication, nuclear DNA is replicated without mitosis or cytokinesis, resulting in cells with DNA content much greater than twice the haploid DNA content (Kudo and Kimura, 2002). Endoreduplication has been studied in some Cactaceae species, like *Mammillaria haageana* (Palomino *et al.*, 1999), *Opuntia* (Palomino *et al.*, 2016) and *Melocactus* (Torres-Silva *et al.*, 2020). In cactus, endoreduplication has been proposed as an adaptive strategy to expand cells for water storage. Flow cytometry analyses in tepals from different developmental stages could be a means to assess if this process is common.

Recent flower developmental studies in *D. speciosus* and *D. eichlamii* showed that one of the main differences between these two species during early development is the initial size of the flower meristem, and later on the size of the ring meristem (Ramírez-Castro *et al.*, 2024). Differences in the size of the flower meristem have been reported as an important factor affecting the number of floral whorls (Bull-Hereñu *et al.*, 2018), but whether meristem size also affects the overall final organ size in *Disocactus* flowers remains unexplored.

Our results could have been affected by two issues: first, we did not collect equivalent developmental stages in each species. Second, the use of pooled tissue (encompassing different tissue and organ types) ended up concealing particular organ-specific processes. Thus, further transcriptomic and gene expression studies based on dissected organs, or, through single-cell transcriptomic approaches, could unravel the particular contributions of the candidate genes described in this work, as well as contribute towards a more ambitious task: to integrate the various pathways that specify tepal organ patterning, size, shape and symmetry into a comprehensive framework. Such a task requires the description and the fine characterization of the underlying genetic and hormonal pathways involved in floral organ development (Varaud *et al.*, 2011).

### Conclusions

To our knowledge, this is the first study in Cactaceae that addresses differential organ growth through the integration of histological and cellular analyses as well as comparative transcriptome data from equivalent developmental stages of floral buds from two species with contrasting flower sizes. We found that while the initial size and number of cells in tepals is similar between *D. speciosus* and *D. eichlamii*, a prolonged proliferation stage in *D. speciosus* coupled with pronounced cell expansion could explain final bud size, which is twice as long as in *D. eichlamii*. While transcriptomes are similar in the two species, differential expression of particular genes involved in cell proliferation and expansion hint at the molecular mechanisms involved.

## Supplementary Material

mcag046_Supplementary_Data

## Data Availability

The data discussed in this publication have been deposited in NCBI's Gene Expression Omnibus and are accessible through GEO Series accession number GSE209808 (https://www.ncbi.nlm.nih.gov/geo/query/acc.cgi?acc=GSE209808).

## SUPPLEMENTARY DATA

Supplementary data are available at *Annals of Botany* online. and consist of the following. Appendix S1: Experimental design of RNA extractions. Appendix S2: Statistical results of cell counts, measurements and tepal length. Appendix S3: Percentage completeness of the core set of genes in BUSCO of the assemblies obtained in this study. Appendix S4: Fraction of variance explained in the two *Disocactus* species. Appendix S5: Maximum likelihood phylogenetic gene tree generated for the *BIG BROTHER* gene. Appendix S6: Maximum likelihood phylogenetic gene tree generated for the *BIG PETAL* gene. Appendix S7: Unigenes involved in the plant hormone signal transduction pathway in *Disocactus*. Appendix S8: Genes that are differentially expressed in the plant hormonal signaling transduction pathways in *D. speciosus* and *D. eichlamii*.
