# Supplementary material for: Insights into the genetic bases of differential organ size in cactus flowers: a case study in two contrasting species
Source: Ann Bot. 2026 Mar 5;138(4):1021–34. doi: 10.1093/aob/mcag046 (PMC13595290; doi:10.1093/aob/mcag046)
Supplement: Supplementary file 1 [file mcag046_supplementary_data.zip › Appendix S1.pdf]

Appendix S1. Experimental design of RNA extractions. We use 3 biological replicates per size, dissecting floral tissue and pericarpel tissue and sequencing separately (6 samples per size). In total 24 libraries were used in the assembly *de novo* (12 samples per species). However, only floral tissue (FL, tepals, stamens and stigma) was analyzed in this study, while pericarpel (PC, pericarpel and carpels) was excluded. Size 1 corresponds to flower buds of 1 cm length; size 2 corresponds to flower buds in pre-anthesis.

| **Species** | **Number of replicates** | **Tissue** | **Size** |
|---|---|---|---|
| *D. eichlamii* | 3 | Floral tissue (FL) | 1 |
| *D. eichlamii* | 3 | Pericarpel tissue (PC) | 1 |
| *D. eichlamii* | 3 | Floral tissue (FL) | 2 |
| *D. eichlamii* | 3 | Pericarpel tissue (PC) | 2 |
| *D. speciosus* | 3 | Floral tissue (FL) | 1 |
| *D. speciosus* | 3 | Pericarpel tissue (PC) | 1 |
| *D. speciosus* | 3 | Floral tissue (FL) | 2 |
| *D. speciosus* | 3 | Pericarpel tissue (PC) | 2 |
