# Supplementary material for: Insights into the genetic bases of differential organ size in cactus flowers: a case study in two contrasting species
Source: Ann Bot. 2026 Mar 5;138(4):1021–34. doi: 10.1093/aob/mcag046 (PMC13595290; doi:10.1093/aob/mcag046)
Supplement: Supplementary file 1 [file mcag046_supplementary_data.zip › Appendix S2.pdf]

Appendix S2. Statistical results of cell counts, measurements and tepal length. DE= *D. eichlamii,* DS= *D. speciosus*. Cell area = μm², Length = cm.

species	tepal_position	variable	n	median	sd
DE	Inner	Cell area	53	1898.459	814.442
DE	Outer	Cell area	144	1817.931	759.932
DS	Inner	Cell area	206	9777.97	3765.247
DS	Outer	Cell area	223	11135.713	4690.1450
DE	Outer	Length	25	1.644	0.1157
DE	Inner	Length	25	1.496	0.1240
DS	Outer	Length	25	4.272	1.055
DS	Inner	Length	25	5.148	0.500
DE	Inner	Number of cells	24	25.5	6.903
DE	Outer	Number of cells	26	35.346	8.555
DS	Inner	Number of cells	25	37.12	6.392
DS	Outer	Number of cells	24	41.146	5.792
