# Supplementary material for: Insights into the genetic bases of differential organ size in cactus flowers: a case study in two contrasting species
Source: Ann Bot. 2026 Mar 5;138(4):1021–34. doi: 10.1093/aob/mcag046 (PMC13595290; doi:10.1093/aob/mcag046)
Supplement: Supplementary file 1 [file mcag046_supplementary_data.zip › Appendix S3.pdf]

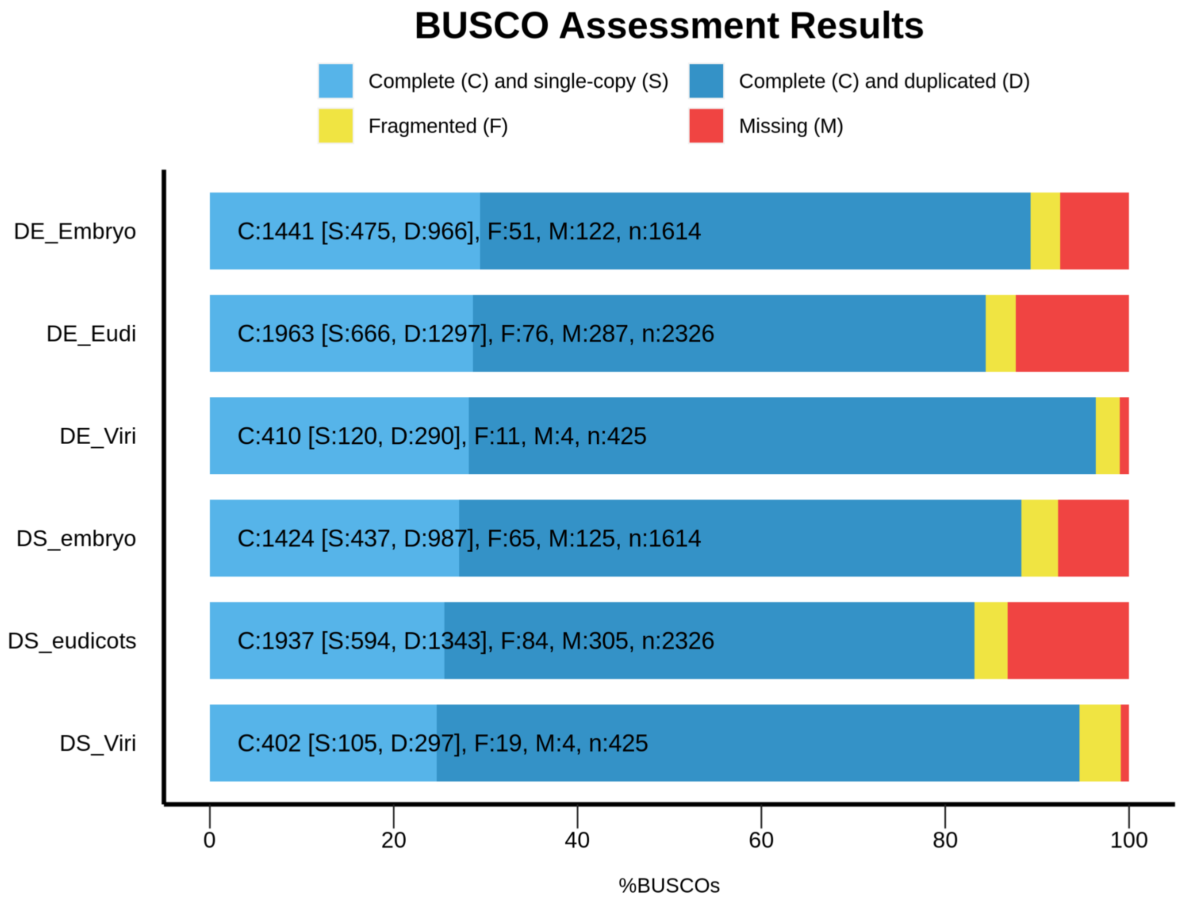

Appendix S3. Percentage of completeness on the core set of genes in BUSCO of the assemblies obtained in this study. BUSCO completeness assessment using Embryophyte database (Embryo), Eudicot database (Eudi) and Viridiplantae database (Vir). DE = *D. eichlamii*, *DS* = *D.speciosus*.
