# Supplementary material for: Insights into the genetic bases of differential organ size in cactus flowers: a case study in two contrasting species
Source: Ann Bot. 2026 Mar 5;138(4):1021–34. doi: 10.1093/aob/mcag046 (PMC13595290; doi:10.1093/aob/mcag046)
Supplement: Supplementary file 1 [file mcag046_supplementary_data.zip › Appendix S4.pdf]

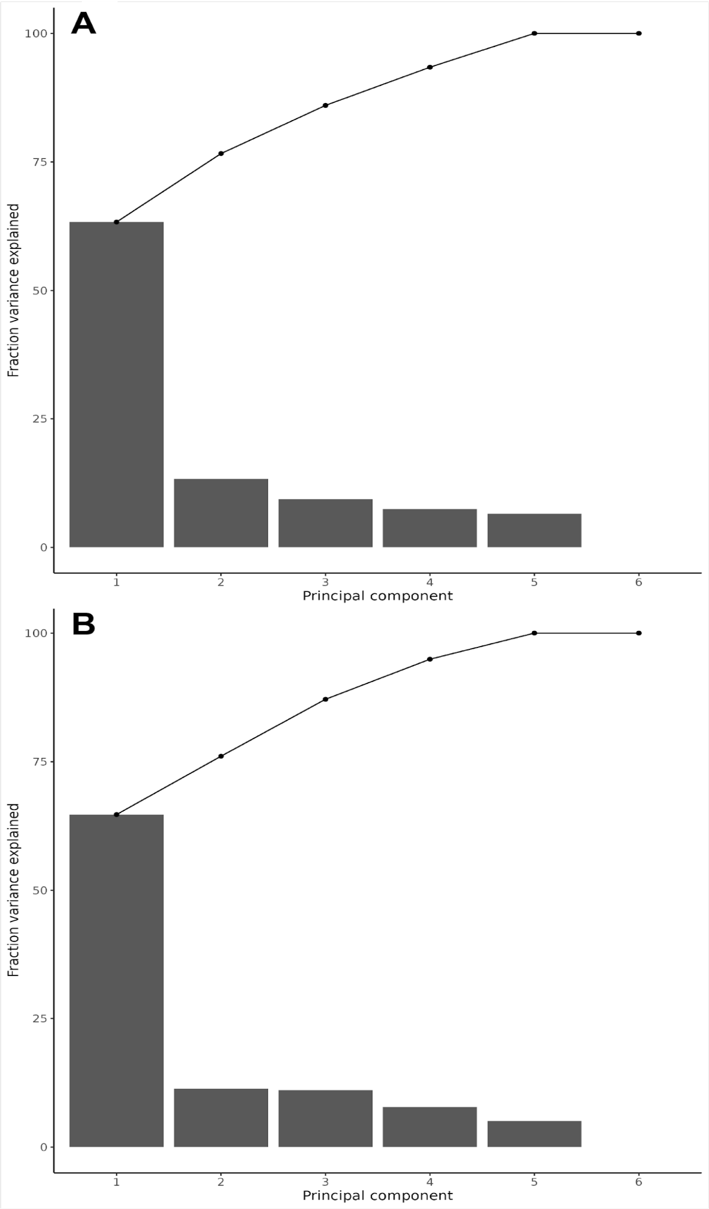

Appendix S4. Fraction variance explained in the two *Disocactus* species. A) Fraction variance explained by the first six principal components of the PCA analysis of all descriptors in *D. speciosus.* B) Fraction variance explained by the first six principal components of the PCA analysis of all descriptors in *D. eichlamii.*
