# Supplementary material for: Insights into the genetic bases of differential organ size in cactus flowers: a case study in two contrasting species
Source: Ann Bot. 2026 Mar 5;138(4):1021–34. doi: 10.1093/aob/mcag046 (PMC13595290; doi:10.1093/aob/mcag046)
Supplement: Supplementary file 1 [file mcag046_supplementary_data.zip › Appendix S5.pdf]

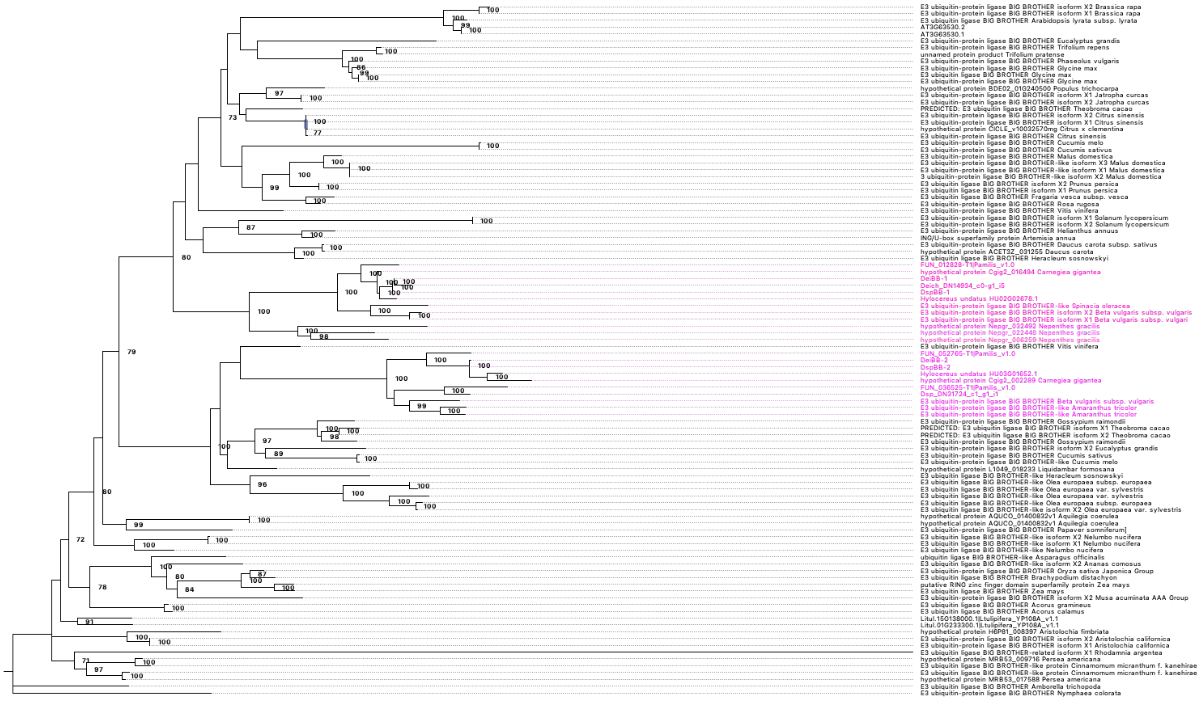

Appendix S5. Maximum likelihood phylogenetic gene tree generated for *BIG BROTHER* gene. The tree shows the relationships between the putative *BB* homologous gene in some representative angiosperm's species. Branches in pink indicate the species included in the Caryophyllales clade. Two copies of the *BB* gene were identified in *Disocactus* which are grouped in two different clades. One copy is more related to *BB* identified in *Arabidopsis thaliana*, which we called *BB-1*; the second copy of *BB*-2, is also present in Caryophyllales but different to the *Arabidopsis BB* gene.
