# Supplementary material for: Insights into the genetic bases of differential organ size in cactus flowers: a case study in two contrasting species
Source: Ann Bot. 2026 Mar 5;138(4):1021–34. doi: 10.1093/aob/mcag046 (PMC13595290; doi:10.1093/aob/mcag046)
Supplement: Supplementary file 1 [file mcag046_supplementary_data.zip › Appendix S6.pdf]

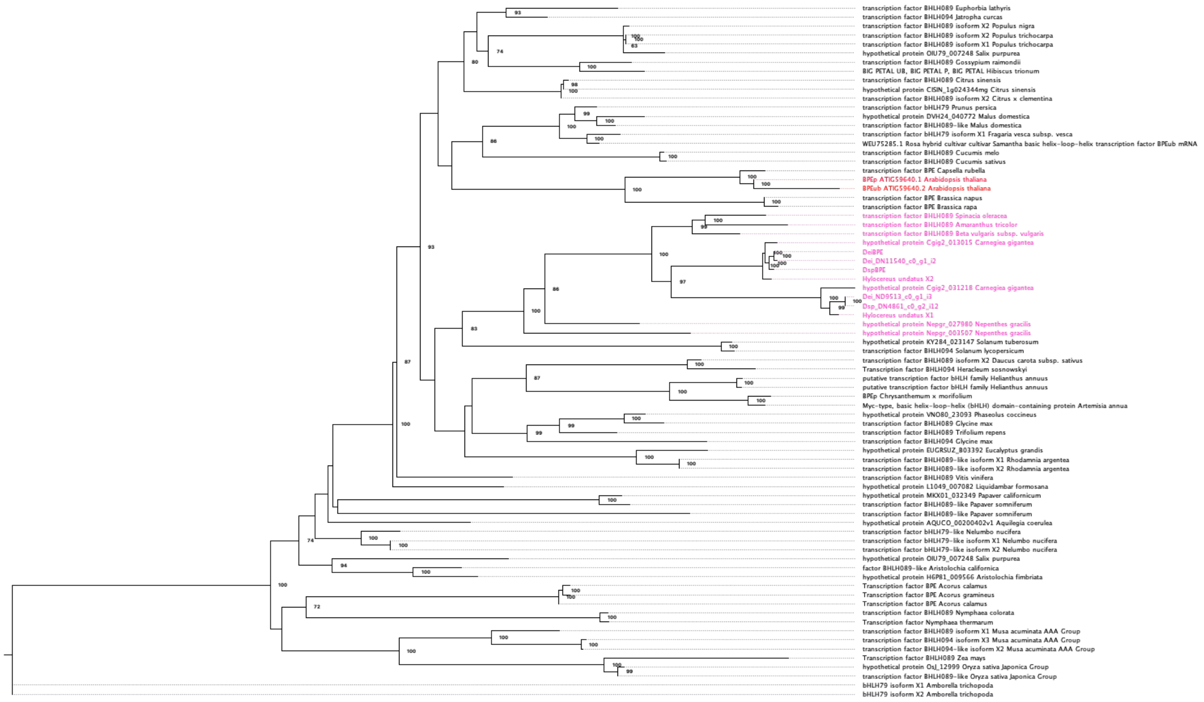

Appendix S6. Maximum likelihood phylogenetic gene tree generated for *BIG PETAL* gene. The tree shows the relationships between the putative *BP* homologous gene in some representative angiosperm's species. In pink are indicated species in the Caryophyllales clade, where *Disocactus* is included. We identified two isoforms of *DsBP* in *D. speciosus*, while three isoforms of *DeBP* were indentified in *D. eichlamii*.
