# Supplementary material for: Insights into the genetic bases of differential organ size in cactus flowers: a case study in two contrasting species
Source: Ann Bot. 2026 Mar 5;138(4):1021–34. doi: 10.1093/aob/mcag046 (PMC13595290; doi:10.1093/aob/mcag046)
Supplement: Supplementary file 1 [file mcag046_supplementary_data.zip › Appendix S7.pdf]

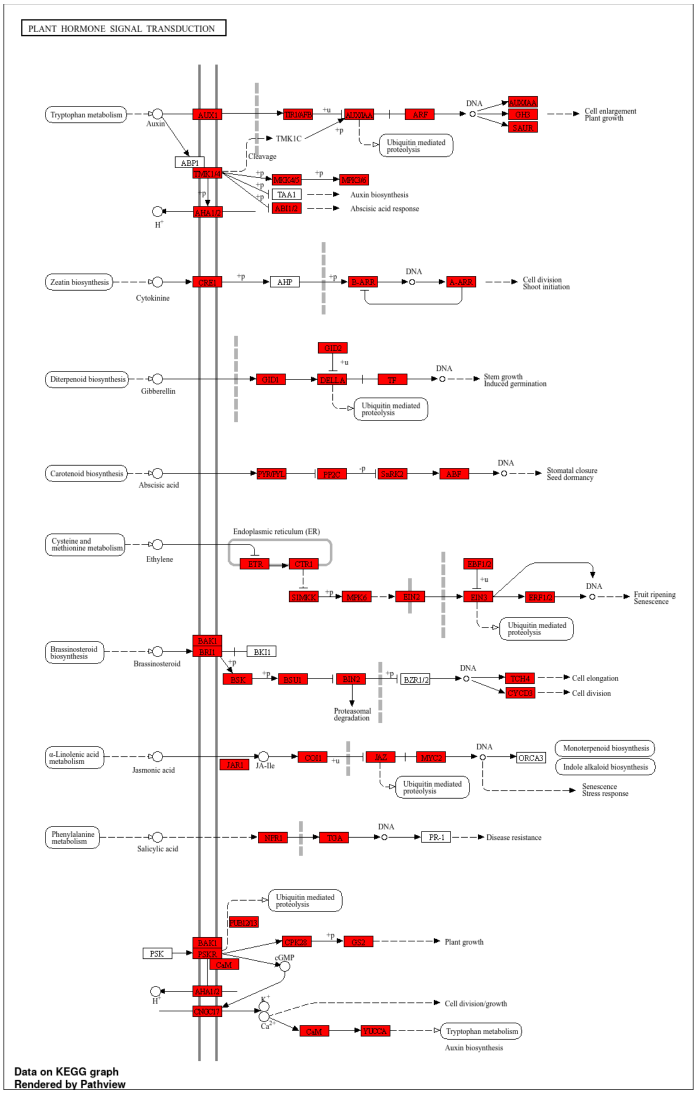

Appendix S7. Unigenes involved in the "plant hormone signal transduction" pathway in *Disocactus*. The genes in red were found by our Illumina sequencing in both species.
