# Supplementary material for: Insights into the genetic bases of differential organ size in cactus flowers: a case study in two contrasting species
Source: Ann Bot. 2026 Mar 5;138(4):1021–34. doi: 10.1093/aob/mcag046 (PMC13595290; doi:10.1093/aob/mcag046)
Supplement: Supplementary file 1 [file mcag046_supplementary_data.zip › Appendix S8.pdf]

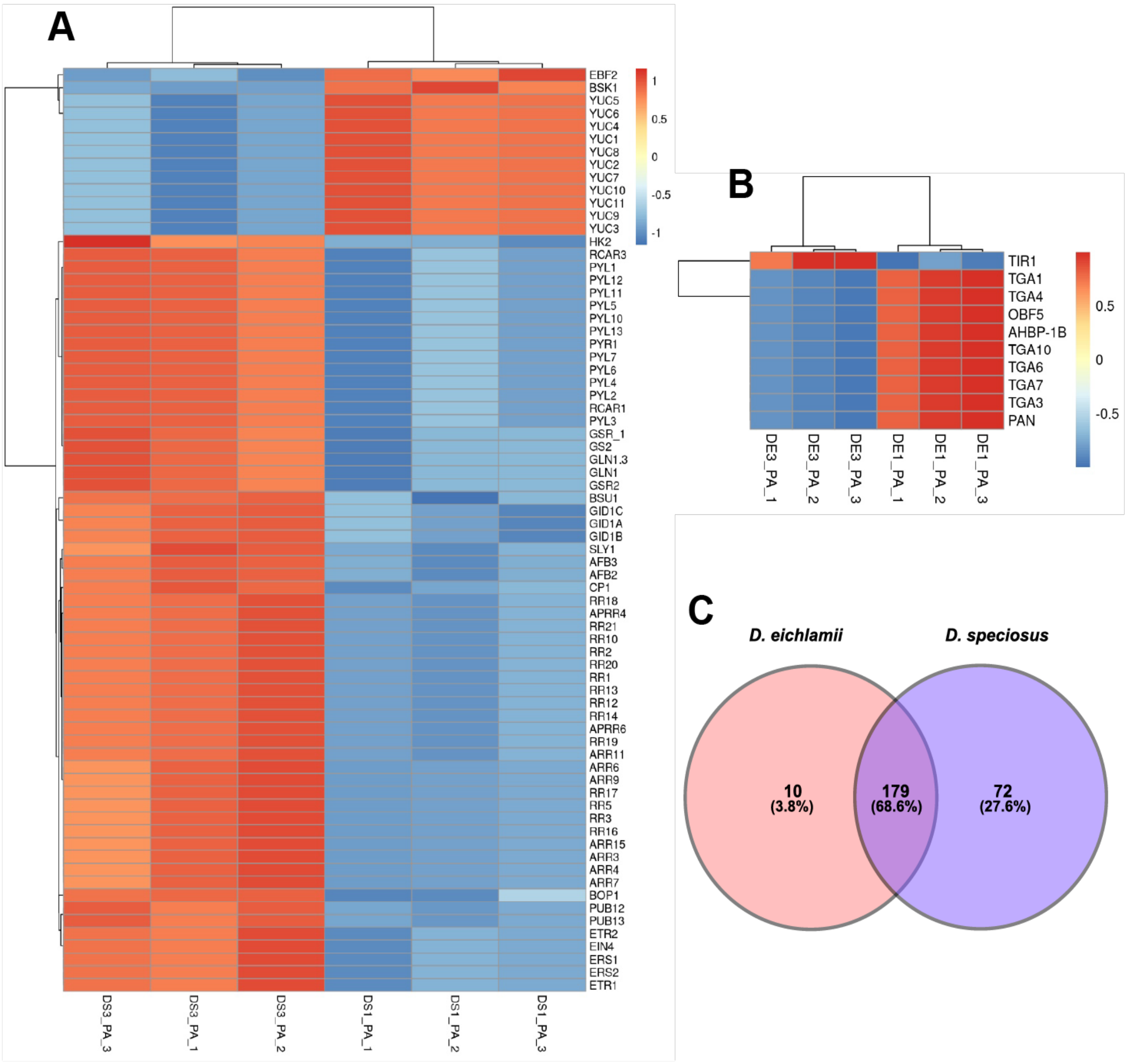

Appendix S8. Genes that are differentially expressed in the "plant hormonal signaling transduction" pathways in *D. speciosus* and *D. eichlamii*. A) Venn diagram. In total 179 DEGs are shared between the two species, while only 10 are differentially expressed in *D. eichlamii* and 72 only in *D. speciosus* B) Genes in the plant hormone signal transduction pathway are only differentially expressed in *D. speciosus*. C) Genes in the plant hormone signal transduction pathway are only differentially expressed in *D. eichlamii*.
